# A 75-Year-Old Female with Hemoptysis and Recurrent Respiratory Infections

**DOI:** 10.1155/2013/832306

**Published:** 2013-04-23

**Authors:** Mary S. Baker, Khalil Diab

**Affiliations:** ^1^Pulmonary & Critical Care Medicine, Division of Pulmonary, Allergy, Critical Care Medicine, and Sleep Medicine, Indiana University School of Medicine, Gatch Clinical Building, Room 260, 541 N. Clinical Dr., Indianapolis, IN 46202-5111, USA; ^2^Clinical Medicine, Division of Pulmonary, Allergy, Critical Care Medicine, and Sleep Medicine, Indiana University School of Medicine, 550 N. University Boulevard, UH 4903, Indianapolis, IN 46202, USA

## Abstract

This paper describes the case of a 75-year-old female who presented with significant hemoptysis over a 7–10 day period. She had a history of a left lower lobectomy 10 years prior for a “lung abscess.” She subsequently had multiple episodes of cough, fevers, and possible pneumonia treated with multiple courses of Amoxicillin and Amoxicillin/Clavulanate. Review of her chest CT upon presentation to the hospital showed a large necrotic lingular infiltrate, which had been progressively increasing in size over at least one year. Bronchoscopy showed a yellowish, soft round body in the superior lingular subsegment. Endobronchial and transbronchial biopsies showed *actinomyces* species. This is a very interesting case of indolent actinomycosis which we suspect had a very slow progressive course secondary to the multiple courses of antibiotics that the patient was treated with.

## 1. Case Report

A 75-year-old female was admitted for further workup of hemoptysis. The hemoptysis started 7–10 days prior to admission and was bright red and significant in volume (often greater than half a cup). The amount increased the day prior to admission. She reported subjective fevers associated with her symptoms. She underwent a bronchoscopy at an outside hospital, which was aborted due to diffuse nonspecific bleeding in the left bronchial tree. She had a history of bronchiectasis and left lower lobectomy in 2002 for lung abscess; culture data from that infection was not available to us. Since the surgery in 2002, she experienced chronic cough and frequent respiratory infections treated with multiple rounds of antibiotics, usually Amoxicillin or Amoxicillin/Clavulanate. 

On physical examination, the patient was afebrile and normotensive. Heart rate was 68 beats/minute, respiratory rate 29 breaths/minute, and oxygen saturation 98% on room air. Head and neck examination showed normal dentition with no lymphadenopathy. Chest examination showed clear breath sounds bilaterally with decreased air entry in the left lower lobe. Abdominal and cardiac exams were unremarkable.

**Figure 1:**
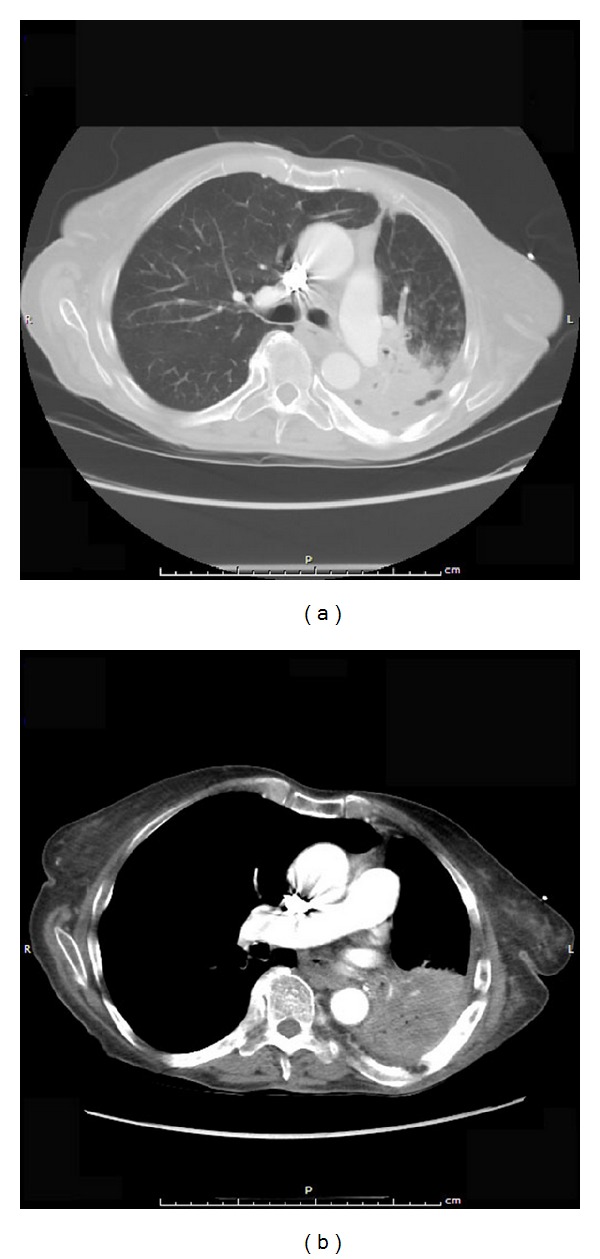
(a) Chest CT with “lung” windows shows an extensive dense lingular infiltrate with scattered air pockets; (b) chest CT with “soft tissue” cuts shows the same lingular infiltrate with extensive necrosis and scattered air pockets.

Pertinent laboratory studies included an arterial blood gas analysis, which showed pH 7.34/pCO_2_ 75 mm Hg/PO_2_ 92 mm Hg/bicarbonate 40.4 meq/L. The chest radiograph showed decreased lung volume in the left base and an extensive lingular and possibly left upper lobe infiltrate. Chest CT is shown in Figures 1(a) and 1(b). Review of her chest CT one year prior showed the same lingular infiltrate with a smaller size. On bronchoscopy, a yellowish, soft round body was identified in the superior lingular subsegment. This body was not immediately recognized. It was removed and sent for histologic analysis. Also, several endobronchial and transbronchial biopsies were obtained. There was subsequent significant and copious bleeding in the lingula, which was treated with intrabronchial epinephrine and resolved. 

Pathology from the retrieved yellowish body revealed actinomyces species. Bronchoscopic cultures did not reveal the diagnosis. After the biopsy results were reported, the thoracic surgery service was consulted. The patient subsequently underwent a left modified Eloesser thoracoplasty. Postoperatively, she did very well. She had complete resolution of her hemoptysis. She also had complete resolution of her chronic cough, which had persisted for ten years. She completed a four-week course of high-dose penicillin G and is being treated with a six-month course of oral amoxicillin. She is currently undergoing planning for a rotational flap to fill in the chest wall defect from the first surgery.

## 2. Discussion

Actinomycosis is a rare but indolent infection caused by anaerobic gram-positive bacteria of the genus *actinomyces*, with the most common pathogen being *Actinomyces israelii*. When first identified, the bacteria was classified as a fungus because of its yellow or whitish mycotic-like appearance. This is what was seen as a whitish body on the bronchoscopy described above. The bacteria make up the normal flora of the oropharynx and are frequently the cause of infection in patients with poor dentition. *Actinomyces* infection results from disruption of mucosal surfaces and can occur anywhere in the body. The classic infection is cervical disease that often presents as a large mass on the jaw or neck. The peak incidence of active infection is reported in the fourth or fifth decades of life. Male predominance exists and most infections involve immunocompetent hosts. Risk factors include alcoholism and poor oral hygiene. Actinomycosis is characterized by a pyogenic response with necrosis and can lead to severe fibrosis [1, 2]. 

Actinomycosis of the respiratory tract often results from aspiration or direct extension of disease from the head or neck. Pulmonary involvement is noted in about 15% of cases. Pulmonary actinomycosis may be complicated by unusual but significant hemoptysis due to parenchymal destruction as *actinomyces* can extend across fissures and even invade the chest wall. Clinically, pulmonary actinomycosis presents like a lung abscess or a nonresolving pneumonia. Patients present with indolent symptoms that evolve over weeks to months. They typically develop a nonproductive cough and low-grade fever, which progresses into a productive cough that is sometimes associated with hemoptysis. Often they will complain of characteristic features of pulmonary infection: fever, cough, sputum production, sometimes night sweats, and weight loss. Chest wall involvement and bony erosion are common. The infection can mimic metastatic disease from lung adenocarcinoma. An uncommon complication of pulmonary actinomycosis is the development of a sinus tract that appears as a bronchocutaneous fistula. Finally, at late stages, patients may manifest clubbing, anemia, and weight loss [1, 2]. 

In our case, we believe the infection evolved over a period of at least several years (although we do not have microbiologic confirmation of that), as we were able to obtain old CT scans of the chest that showed a progressively enlarging lingular necrotic area. Our patient had a chronic productive cough and low-grade fevers and was treated for pneumonia with multiple rounds of antibiotics. We believe that the antibiotics may have acted to slow the progression of this indolent infection. 

Patients with indolent *actinomyces* infections that evolved over a period of 9 months to several years are shown in Table 1.

Diagnosis can be difficult as these bacteria are part of the normal flora and are often difficult to culture. There are currently no serologic tests that can be used for diagnosis. Bronchoscopy is often not diagnostic unless endobronchial disease is present. Bronchial washings and brushings are not helpful. Transbronchial or open lung biopsies are often in order to make the diagnosis. With the propensity of actinomycosis to mimic malignancy, diagnosis is often made after surgical resection [3]. 

Untreated infections will ultimately result in death, but if treatment is initiated early the rates of cure are greater than 90%. Prolonged treatment is needed, most commonly with penicillin as the drug of choice. Usually a combination of high doses of intravenous and oral antibiotics is needed. Alternative antimicrobial agents include tetracyclines, macrolides, and chloramphenicol. Surgery is indicated for bulky disease or necrotizing infections that have eroded through tissue, vasculature, or parenchyma. Relapse is common, but with treatment, long-term prognosis is good. The combination of surgery plus antibiotics has been shown in small trials to prevent relapse [3, 4]. 

It is likely the patient was spared fulminant invasive disease into her chest wall by the intermittent doses of amoxicillin she received for her recurrent pneumonias, which possibly served to suppress her *actinomyces* infection and delay her diagnosis.

In conclusion, pulmonary involvement in actinomycosis can mimic the presentation of lung cancer and can result in massive hemoptysis and life-threatening disease if left unchecked. Early detection is important, but diagnosis is difficulty; therefore, a high level of suspicion is needed.

## Figures and Tables

**Table 1:** Indolent cases of actinomycosis.

Source	Publication year	Age or number of patients	Symptoms	Hospital presentation	Diagnosis	Radiologic findings	Treatment	Outcome
Reechaipichitkul et al. [5]	2005	41 years	Fevers & hemoptysis over 2 years	Massive hemoptysis	Histopathology	Extensive right upper lobe infiltrate	Emergent right upper lobectomy, intravenous augmentin followed by amoxicillin	Hemoptysis resolved
Ma et al. [6]	2009	66 years	Fevers & productive cough over 4 years	Indolent symptoms of fever and cough	Histopathology	Right middle lobe infiltrates	Surgical resection and antibiotics	Symptoms resolved
Dujneungkunakorn et al. [7]	1999	16 patients	Cough and hemoptysis most common; mean duration of symptoms: 9 months	Indolent symptoms as reported	Histopathology	Mass-like shadowing most common (37%)	Surgical resection in 8 patients; antibiotics for all	All patients who had surgical resection were cured; 20% of antibiotic-only group did not respond
